# Socioeconomic status, linguistic skills and language background differentially relate to preschoolers’ emotional and behavioural profile

**DOI:** 10.1186/s13034-024-00823-y

**Published:** 2024-10-18

**Authors:** Paola Bonifacci, Viola Ravaldini, Martina Cangelosi, Valentina Tobia

**Affiliations:** 1 Department of Psychology, University of Bologna, Viale Berti Pichat 5, 40127 Bologna, Italy; 2 Faculty of Psychology, University Vita-Salute San Raffaele, Via Olgettina 58, 20132 Milan, Italy; 3 IRCCS San Raffaele Hospital– Ville Turro, Via Stamira d’Ancona 20, 20127 Milan, Italy

**Keywords:** Language, Socioeconomic status, Heritage bilinguals, Emotions, Peer relationships, Prosocial behaviour

## Abstract

**Background:**

Proximal and distal factors interact to shape children’s development and well-being. The present study aimed to examine socioeconomic status (SES), linguistic skills, and language background as concurrent predictors of socio-emotional and behavioural outcomes in heritage bilingual and monolingual children attending preschool.

**Methods:**

Parents of 1810 children (mean age = 63.42 months ± 7.36), attending preschool in Italy, completed the Four Factor Index of SES and the Strengths and Difficulties Questionnaire (SDQ). Teachers (*n* = 99) completed a questionnaire on children’s linguistic skills and emotional and behavioural profiles. A subsample of 995 children was administered an expressive vocabulary task in Italian.

**Results:**

Regression analyses showed that linguistic skills were the only concurrent predictor of conduct problems, as well as the dominant predictor of hyperactivity/impulsivity, peer problems, and better prosocial behaviour. SES was negatively related to ADHD traits, peer problems, and prosocial behaviour. Finally, heritage bilingualism background was associated, although not as a primary predictor, with increased emotional problems, peer relationship problems, and lower teacher-rated emotional and behavioural skills. However, it was the main factor positively associated with prosocial behaviour.

**Conclusions:**

The implications of these findings for research in this area and for educational policy are discussed, highlighting the need for a multidimensional perspective that includes linguistic skills and SES in the assessment of children’s emotional and behavioural outcomes.

## Background

Multidimensional models clearly suggest that environmental (distal) factors interact with individual (proximal) factors in shaping children’s developmental trajectories [1, 2]; linguistic skills and socioeconomic status (SES) have been widely examined in the literature as potential protective (or risk) factors for children’s emotional and behavioural outcomes. Linguistic skills have been found to be related to emotional and behavioural development in typical children [3] and atypical (i.e., with developmental language disorders) populations [4, 5]. More in-depth studies have then explored these trends even in bilingual populations (i.e., children from families with a migratory background) [6]. SES has been found to underlie children’s discrepancies in academic achievements [7–10], linguistic skills [11–13], and socio-emotional well-being [14, 15]. However, SES is better viewed as a structural variable rather than an intrinsic characteristic of the family/individual itself [16] and the impact of SES may be modulated by other social and family characteristics [17, 18]. In addition, some meta-analyses [19] found diminished, although significant, effect sizes of the relationship between SES and academic achievement than suggested by previous studies [20], with the effect size being more robust in more economically developed countries [21] and in younger children compared to older ones [22]. Finally, the definition and operationalization of SES is still a matter of debate and results might depend on how it is conceptualized and measured [23].

Following globalisation and migration routes, many children attending school in Western countries live in families with a migratory background and are often exposed to one or more heritage languages within the family. For the purpose of the present study, the term “heritage bilinguals” (HB) will be adopted in line with current literature [24], although plenty of different labels are available in previous studies [25]. In preschool years, HB may have lower second language (L2) proficiency, compared to monolingual peers, given to the still limited exposure to the societal language [24], and they might experience socio-economic disadvantage. In Italy, where the present study has been carried out, the index of absolute poverty is 35.6% for families with both parents holding non-Italian citizenship compared to 6.4% of families with Italian citizenship [26]. The present study focuses on understanding the differential contribution of SES and language proficiency as concurrent predictors of children’s socio-emotional and behavioural outcomes in a broad sample that includes Italian monolingual children and HB, thus taking into account family language background as an additional distal factor.

### The relationships between language skills and socio-emotional well-being

Although language and emotional/behavioural difficulties often co-occur, the direction of the relationship is harder to define [27]. Some authors suggest a bidirectional relationship with reciprocal influences between the two [28], whereas others support unidirectional pathways as being more likely, with language difficulties as a prominent factor leading to socio-emotional and behavioural difficulties [29, 30]. In this line, [31] reported that the main predictor of children’s well-being was vocabulary growth from 4 to 9 years rather than vocabulary knowledge at four years. A study by Bichay-Awadalla et al. [32] on preschoolers reported, instead, different patterns of relationships with socio-emotional development according to different aspects of linguistic skills, with bidirectional patterns for expressive skills and unidirectional patterns for receptive skills. Other perspectives suggest the possibility that the relationship between linguistic skills and emotional and behavioural problems might be mediated by other (third) factors or that behavioural and emotional problems might hinder full language development [27].

Some studies have also explored these trends in HB children. Specifically, the literature has found associations between problematic behaviours and lower linguistic competence in preschool bilingual children [6, 33–35], with greater externalising behaviours in primary school children with lower L2 linguistic skills [36, 37].

There may be adverse factors related to the migratory process of some families, such as interrupted education, poverty, frequent transitions, exposure to violence, and separation from family, that might increase vulnerability [38–40]. Therefore, language background shouldn’t be considered only in terms of language competence but as a more complex variable that includes the potential impact on the child’s well-being of speaking a language at home that’s different from the one employed in social contexts. The perception that the heritage language is devalued or under-recognised as a linguistic ability both at school and in social contexts could affect the family climate and, consequently, the child’s well-being. In line with this, some authors reported an increased risk for emotional and behavioural problems in HB children and adolescents [41], although some studies did not report differences in prosocial behavior and social skills between HB and monolingual peers [42]. On the other hand, positive attitudes towards multilingualism and good connections with heritage language and culture could foster enriched social inclusion, emotional well-being and family cohesion [9, 43–47].

### The relationships between socioeconomic status and children’s verbal, emotional and behavioural skills

The impact of SES on language and socio-emotional outcomes could be explained by multiple mechanisms. On the one side, families from low socioeconomic backgrounds may have less access to materials and information, social resources and educational opportunities (e.g., exposure to books, reading practice, quality of schools, etc.) to support children's verbal and emotional development [48]. In addition, the availability of material resources may affect subjective experience and how individuals shape their experiences and behaviors (e.g., fewer resources may be associated with an increased focus on external social forces and other individuals that influence one's life outcomes [49]. Some data from the literature suggest that SES has a direct impact on children’s linguistic skills: for example, vocabulary knowledge and vocabulary growth, syntax and language learning process [12, 13, 50], as well as literacy skills [51, 52] are known to be negatively affected by lower levels of SES. However, some authors [8], also evidenced that some additional factors (i.e., higher country SES and alphabetic languages) might induce a larger indirect impact from SES to reading ability. Also, further evidence suggests that other variables such as parental participation and school resources [53] may buffer the SES-academic achievement relationship. A large and recent study [54] found that SES (operationalized as maternal education) and multilingualism were not significantly associated with children’s verbal production over the first 4 years of life, but other variables related to the home environment, such as the amount of adult talk, were predictors of children’s linguistic skills. It should be considered that previous studies have found high variability within both low- and high-SES families in home-related variables such as their home support for learning (i.e., home literacy/numeracy) [7, 55, [56]. Evidence has been collected on the relationship between SES and verbal abilities in HB populations. SES has been found to be related to HBs’ L2 vocabulary size [57, 58], productive and receptive morphosyntactic skills [59, 60] and discursive-semantic abilities [61] in the latter study, it was also observed that the greater the cumulative exposure to school language, the stronger the correlation between SES and sentence repetition score.

SES might also have an impact on children’s socio-emotional and behavioural development [62] and has been found to be linked to the development of psychopathology and lower academic achievement [10, 63–65]. In a meta-analysis [66], it was found that SES was more strongly related to externalising rather than internalising symptoms, but it emerged that the relationship between SES and child psychopathology is likely to vary in different populations of children and in different communities. Furthermore, some authors reported a weak association between SES and psychopathological vulnerability in some ethnic groups [67, 68]. The association between SES and psychopathological indicators, therefore, is still a matter of debate, particularly for HB children.

### Present study

Based on previous literature and theoretical multidimensional models, multiple relationships emerge among SES, linguistic development, language background, and emotional and behavioural development. However, there is a need for more extensive research that accounts for the influence of both proximal and distal factors on children’s outcomes, particularly for the emotional and behavioural dimensions. As evidenced by Singh and Rajendra [69] greater attention to socioeconomic status in developmental research is still needed to increase the generalizability of data in psychological research. This is particularly the case when studying specific populations, as there is a risk of biased inferences if both proximal and distal factors are not accounted for [24]. Finally, most of the previous work has been conducted in English-speaking contexts, and there might be cultural differences that limit the generalisability of results from one context to another. It is therefore necessary to develop studies in different cultural contexts, and the present study, conducted in Italy, may represent an important contribution to the previous literature.

The aim of the present study is to investigate, with a multi-informant (parents, teachers) and multi-method (questionnaires, tests) approach, differences and similarities in linguistic and socioemotional/behavioural skills between HB and monolingual peers attending preschool, as well as the role of SES, linguistic skills and language background as concurrent predictors of socio-emotional and behavioural skills in HB children and monolingual peers.

The main research question of the present study is: what is the association of linguistic background, SES and linguistic skills on socioemotional and behavioural skills in HB and monolingual peers?

Given that previous literature highlighted influences of linguistic background, linguistic skills and SES on socioemotional and behavioural skills, we wanted to analyse the differential role of linguistic background, SES and linguistic skills, considered together, on children’s socioemotional and behavioural skills. We expect SES [10, 62–66] and linguistic skills [29, 30, 32] to be the main predictors of children’s well-being, and once accounted for these variables, to observe a marginal role of HB per se.

A preliminary analysis of group differences between HB and monolingual peers was also conducted to: (a) report what the analysis of group differences between HB children and monolingual peers in socio-emotional and behavioral skills might suggest when SES and language skills are not taken into account. This type of comparison runs the risk of overestimating differences based on language background alone; (b) to justify the inclusion of the dichotomous (HB vs. monolinguals) group variable in the regression models addressing the main research question.

## Method

### Participants

The study involved a community sample of 1810 children attending the second and third year of 58 public all-day preschools, located in Bologna, a city in northern Italy. Participants were recruited as part of a larger project aimed at enhancing language skills in preschoolers in collaboration with the Municipality of Bologna; a letter was sent to parents with informed consent and questionnaires, participation was voluntary. Children whose parents provided consent were administered the vocabulary task and data from the teacher questionnaire were entered. The teachers completed the questionnaire on individual children as an action of the larger project. Within this sample, 1261 children were from families speaking mainly Italian in the family context (monolinguals), and 549 were HB children. The children were classified as HB if the parents reported in the questionnaire that they spoke a Heritage Language in the family context. Further, most families had a migrant background, with one parent (29.3%) or both (59.4%) born in another country, whereas 11.3% had both parents speaking a heritage language in the family but were born in Italy. For more extended demographic details see Table 1. Socioeconomic status was assessed through the Hollingshead Four Factor Index of Social Status (see Materials section).

**Table 1:** Demographic information

	Total sample	Monolinguals	HB children
Number of children	1810	1261	549
Percentage of females	48.9%	49%	49%
Mean age (months)	63.42 ± 7.36	63.52 ± 7.39	63.18 ± 7.29
SES	39.34 ± 14.15	43.65 ± 12.44	29.43 ± 12.80
Background	-	Italian speaking families	Heritage language speaking families
Parents born in another country	-	-	One parent: 29.3%, Both parents: 59.4%
Both parents born in Italy	-	-	11.3%
Main languages spoken	-	-	Arabic: 18.6%, Romanian: 18.2%, Pidgin English: 8.6%, Albanian: 7.8%, Bengali: 7.3%, Spanish: 7.3%, Urdu: 5.8%, Russian: 4.4%, African French: 2.2%, Tagalog: 3.3%, Chinese: 1.8%, Portuguese: 1.6%, Ukrainian: 1.5%, Sinhali: 1.4%, Greek: 1.3%, Polish: 1.3%, Others: 7.6% (all < 1%, e.g., Serbian, Tamil, Bulgarian, Somali, etc.)

A few children (*n* = 19) had a certified mild disability. All parents filled in the study questionnaire (see below for details). Then, for each child, a teacher filled in a questionnaire, for a total of 99 teachers involved. Teachers filled in the questionnaire for a variable number of children depending on how many families adhered to the study in each class. The Vocabulary task (see below for details) was administered to a subsample of 995 children, of which 31.76% are in the HB group. The Italian preschool system involves children aged from 3 to 6 years old and is structured as follows: a three-year program during which no formal teaching of literacy is provided. However, the children might be engaged in activities to improve their language skills in playful activities.

### Materials

#### Questionnaires for parents

##### Socio-economic status (SES)

Parents completed the Hollingshead Four Factor Index of Social Status. To achieve a composite score for each child’s SES, information regarding both parents’ educational level and occupation was scored from 1 to 7 for educational level and 1 to 9 for occupation. Then, SES scores for each parent were calculated using the formula (educational level*3 + occupation*5); the mean between parents’ SES was used as the child’s SES. In the case of single parents, their unique score was used. The minimum and maximum scores ranged from 8 to 66. Scores between 8 and 29 are considered low-medium SES, scores between 30 and 30 are defined and medium SES and scores above 40 are considered medium-high SES.

##### Strengths and difficulties questionnaire

The single-sided version of the SDQ-parents [70] was administered. The SDQ is available in many languages and parents were offered the possibility to access their preferred language when requested. The questionnaire has been found to be concordant for both native and immigrant parents [71]. This questionnaire includes 25 items describing positive and negative behavioural traits; respondents use a 3-point Likert-type scale (0 = not true, 1 = somewhat true, and 2 = certainly true) to rate each item referring to the son/daughter. The 25 items are divided among the following five scales (Cronbach alpha reliability scores are calculated on the study sample): Emotional Symptoms (α = 0.66), Conduct Problems (α = 0.53), Hyperactivity-Inattention (α = 0.70), Peer Relationship Problems (α = 0.60), Prosocial Behaviour (α = 0.65). A higher score corresponds to more severe difficulties on the four scales describing negative behaviours. On the Prosocial Behaviour scale, a higher score indicates more positive behaviours. For each scale, the maximum score is 10. The observed reliabilities show poor to acceptable values [72], in line with previous studies investigating psychometric properties of the SDQ-parents in Italian samples of older students [73, 74]. 

#### Questionnaire for teachers

Children’s early linguistic skills (in Italian) and emotional-behavioural profile were assessed with a proxy-report questionnaire administered to their teachers. The whole questionnaire consists of 20 items [18, 75, 76], developed based on the early cognitive, literacy, numeracy, and behavioural skills deemed adequate for preschoolers based on the Italian curriculum and the previous literature. The questions were first qualitatively validated by groups of teachers who provided feedback on the items’ clarity. For each item, the name of the competence was accompanied by a short definition and some examples (e.g., *phonological awareness*: “It refers to the child’s ability to perform fusion/segmentation tasks, such as splitting or joining the pieces of the word banana: ba-na-na). For the aim of the present study, we considered only two subscales:

- Linguistic skills (Five items: phonological awareness, morphosyntactic comprehension and production, narrative skills, pre-writing skills);
- Behavioural profile (Four items: the ability to respect waiting time, sociality, emotional resources, interest in activities).

The teachers rated their evaluations of the children’s skills on a five-point Likert scale from “never/absent” to “always/excellent competence”. The Cronbach’s alpha for the scales is 0.904 for the linguistic area and 0.968 for the behavioural area.

##### Children’s expressive vocabulary

The early language skills of the children were assessed through the expressive vocabulary task, administered in Italian, from the Learning Difficulties Indexes– IDA [18]. In this task, children were asked to name 36 images disposed on three grids, with 12 images each selected for decreasing frequency in spoken language [77]. The accuracy score, ranging from 0 to 36 (1 point for each correct answer), was considered. The Cronbach’s alpha of the scale was 0.85, according to the test manual.

### Procedure

Questionnaires on SES and SDQ were provided to parents as paper and pencil questionnaires. Parents could complete it together or by who spends more time with the child, usually the mother. The teachers were required to complete the questionnaire for each child within one month to allow them to observe the children’s linguistic skills and emotional and behavioural skills. Early language tasks in Italian were administered individually by a trained psychologist in a quiet room at the children’s school in a single session lasting about 10 min. Special attention was given to ascertaining children had correctly understood the instructions. Participants involved in the study gave informed consent, the study was conducted in accordance in accordance with the Declaration of Helsinki and the University of Bologna Bioethical Committee approved the project (Prot. 322431, December 21, 2021).

### Data analysis

We ran the analysis using the program RStudio 1.0.153 [78]. We used the “lmtest” [79] and “car” [80] for our main analyses. As preliminary analyses, Pearson correlation analyses among the main variables of the study are reported, with the aim of examining the relationships between the key variables in our study. For this purpose, we used the “hmisc” package [81]. Then, group differences (monolinguals vs. HB) across the main measures considered were verified through Welch Two Sample t-tests, which are particularly robust when there is high variability within a sample (i.e., both for socioeconomic and linguistic background).

The second set of models aimed to understand how language background (dichotomic variable), together with SES and verbal knowledge (continuous variables), related respectively to parents’ and teachers’ evaluation of children’s socioemotional and behavioural skills (measured through SDQ and ad-hoc teacher questionnaire respectively). Given the high correlation between the vocabulary task and the assessment of linguistic skills through teachers’ questionnaire (*r* =.550), we decided to include in the analyses only the latter variable to avoid collinearity and consider a wider sample, therefore reported results are referred to the sample size of 1810 participants. Furthermore, we ran dominance analyses for every model. Indeed, this analysis has been used to determine the relative importance of every predictor inserted in our regressions. The “relaimpo” package [82] has been employed for this purpose.

## Results

### Preliminary analysis: correlations and group differences

Correlations among the main variables of the study are reported in Table 2, group differences are reported in Table 3.

**Table 2:** Correlations among the main variables of the study

	SES	1	2	3	4	5	6	7
Vocabulary^ (1)	0.436**							
Linguistic skills (TE)° (2)	0.377**	0.550**						
Emotion & Behaviour (TE)° (3)	0.168**	0.253**	0.665**					
SDQ - Emotional symptoms (4)	− 0.107**	− 0.092**	− 0.079**	-0.042				
SDQ - Conduct Problems (5)	− 0.061**	− 0.075*	− 0.095**	− 0.186**	0.277**			
SDQ - Hyperactivity/inattention (6)	− 0.185**	− 0.128**	− 0.206**	− 0.272**	0.236**	0.423**		
SDQ - Peer Relationship Problems (7)	− 0.237**	− 0.238**	− 0.239**	− 0.144**	0.507**	0.199**	0.191**	
SDQ - Prosocial Behaviour	− 0.090**	-0.039	0.049*	0.111**	− 0.247**	− 0.343**	− 0.287**	− 0.273**

**p <. 01; * *p* <.05

^ *N* = 955, °TE = Teacher Evaluation

**Table 3:** Welch two sample T-tests

	Subscale	HB (Mean)	Monolinguals(Mean)	t	df	*p*	95% CI (Lower)	95% CI (Upper)
SES		29.426	43.654	-21.929	1016.5	< 0.001	-15.501	-12.955
Linguistic skills		3.518	4.217	-13.534	835.32	< 0.001	-0.800	-0.598
SEB Skills^		3.769	3.990	-4.744	931.15	< 0.001	-0.314	-0.130
SDQ	Emotions	2.047	1.641	4.241	966.9	< 0.001	0.218	0.593
	Conduct	1.677	1.642	0.451	1005.1	ns	-0.119	0.190
	ADHD	3.718	3.270	4.060	1099.4	< 0.001	0.231	0.663
	Peer Relations	2.124	1.250	10.075	984.18	< 0.001	2.124	1.250
	Prosocial Behaviour	8.179	7.789	4.443	1110.5	< 0.001	8.179	7.788

The table displays the results of Welch Two Sample T-tests comparing the means of HB and Monolinguals for each variable, with its subscales. p-values less than 0.05 are considered statistically significant

^Socioemotional and behavioural

As it can be observed from Table 3, HB belonged to an averagely lower socioeconomic background compared to monolinguals and performed significantly worse in the vocabulary task, in linguistic skills, and in the behavioural profile according to the teacher’s evaluation. Concerning SDQ Scales, except for the behavioural scale, bilingual children had significantly higher rates of reported difficulties in all domains, as reported their parents’ answers to the questionnaire. However, they also had higher values on the Prosocial Skills Scale, meaning that, according to their parents, they tend to be more prosocial in comparison with their monolingual peers. Since these comparisons did not take into account the role of SES and L2 linguistic skills further models were developed to better investigate if these differences based on language background hold when other variables are taken into account.

### Concurrent predictors of socio-emotional and behavioural profile

All results of the regression analyses are presented in Table 4. For each analysis, language background, linguistic skills and SES were considered predictors and, in turn, SDQ subscales and teacher ratings were considered as dependent variables.

**Table 4:** Results of the regression models on SDQ subscales and teacher ratings

	β	Std. Error	*t* value	Pr(>|t|)	Dominance
*Model on SDQ Emotional Problems*					
(Intercept)	2.522	0.190	13.244	< 0.001	
Language background	0.240	0.106	-2.264	< 0.05	0.006
Linguistic skills	-0.064	0.048	-1.319	0.188	0.003
SES	-0.008	0.004	-2.421	< 0.05	0.007
*Model on SDQ conduct problems*					
(Intercept)	2.329	0.159	14.624	< 0.001	
Language background	0.135	0.089	1.524	0.127	0.001
Linguistic skills	-0.144	0.041	-3.556	< 0.001	0.008
SES	-0.005	0.003	-1.678	0.093	0.003
*Model on SDQ Hyperactivity/Inattention scale*					
(Intercept)	5.650	0.227	24.887	< 0.001	
Language background	0.112	0.126	0.888	0.375	0.003
Linguistic skills	-0.374	0.058	-6.479	< 0.001	0.031
SES	-0.021	0.004	-5.005	< 0.001	0.022
*Model on SDQ Peer relationship problem scale*					
(Intercept)	3.479	0.171	20.347	< 0.001	
Language background	0.485	0.095	-5.089	< 0.001	0.031
Linguistic skills	-0.265	0.044	-6.093	< 0.001	0.034
SES	-0.014	0.003	-4.557	< 0.001	0.030
*Model on SDQ Prosocial behaviour scale*					
(Intercept)	7.753	0.185	41.891	< 0.001	
Language background	0.383	0.103	-3.720	< 0.001	0.009
Linguistic skills	0.212	0.047	4.512	< 0.001	0.007
SES	-0.011	0.003	-3.202	< 0.01	0.007
*Model on teachers’ evaluation of socioemotional and behavioural skills*					
(Intercept)	1.578	0.068	23.187	< 0.001	
Language background	-0.182	0.038	-4.818	< 0.001	0.009
Linguistic skills	0.653	0.017	37.747	< 0.001	0.434
SES	-0.004	0.001	-2.928	< 0.01	0.014

#### Emotional symptoms

The categorical variable Language Background was significant (*p <*.05, β = 0.240), meaning that bilinguals have averagely higher scores of emotional symptoms according to parents’ reports. The continuous variable L2 linguistic skills was not significant (*p* =.188), while SES was significant (*p* <.05, β = − 0.008), meaning that children coming from higher socioeconomic status families show overall less emotional problems. The dominance analysis reveals that SES’ contribution seems to be particularly relevant in the present model.

#### Conduct problems

The categorial variable Language Background was not significant (*p =*.127).

The continuous variable linguistic skills was significant, with *p* <.001, with a negative estimate β = -0.144, meaning that children with higher performance in the verbal domain show fewer conduct problems. On the contrary, SES was not significant, with a *p =*.093. The dominance analysis reveals that linguistic skills contribution seems to be particularly relevant in the present model.

#### Hyperactivity/Inattention scale

The categorial variable Language Background was not significant (*p =*.375). The continuous variables L2 linguistic skills and SES were both significant, with *p <*.001, β = -0.374 and β = -0.020, meaning that children with higher performance in the verbal domain in Italian show fewer hyperactivity/inattention problems, as well as children coming from higher SES families. The dominance analysis reveals that the contribution of L2 linguistic skills emerges as particularly significant in the current model, demonstrating a stronger and more influential association compared to the other variables examined.

#### Peer relationship problems

The categorial variable Language Background was significant (*p <*.001) with a positive estimate (β = 0.485). This result indicates that HBs have average higher peer relationship problems compared to monolinguals. The continuous variables L2 linguistic skills and SES were both significant with *p* <.001, β = − 0.265 and β = − 0.014, meaning that children with an averagely better performance in L2 linguistic skills, as well as children coming from higher socioeconomic status families, show overall fewer peer relationships problems. The dominance analysis indicates that L2 linguistic skills appear to be notably significant within the present model, suggesting a pronounced influence compared to other variables under investigation.

#### Prosocial behaviour scale

The categorial variable language background was significant (*p <*.001) with a positive estimate (β = 0.383). This result means that bilinguals have an average higher prosocial behaviour. The continuous L2 linguistic skills and SES were both significant with a positive estimate for linguistic skills (*p* <.001, β = 0.212) and a negative estimate for SES (*p* <.01, β = − 0.011), meaning that children with better L2 linguistic skills have better prosocial behaviour, but those coming from higher socioeconomic status families show lower prosocial behaviour skills. The dominance analysis underscores the notable significance of bilingualism’s contribution within the current model, indicating a discernible role that surpasses that of other variables under examination.

#### Teachers’ evaluation on children’s socioemotional and behavioural skills

Concerning the model on teachers’ evaluation of children’s socioemotional and behavioural skills, language background turned out to be significant, with *p* <.001 and β = -0.183, meaning that HBs received by teachers lower scores in socioemotional and behavioural skills. L2 Linguistic skills and SES also turned out to be significant, respectively with *p* <.001, β = 0.653 and *p* <.01, β = -0.004, indicating that children with higher linguistic skills are evaluated as significantly better in terms of socioemotional and behavioural skills. On the contrary, a higher SES was associated with lower scores in terms of socioemotional and behavioural skills. The dominance analysis highlights the substantial relevance of linguistic skills within the current model, indicating a pronounced influence that exceeds that of other variables under investigation.

## Discussion

The present study investigated the differential contribution of language background (monolinguals vs. HB), SES and linguistic skills on children’s emotional and behavioural profiles.

A preliminary analysis of group differences between HB children and monolingual peers was conducted in order to justify the inclusion of a dichotomous variable (language background) in the regression models and to highlight the risk of overestimating differences based on language background alone if other variables such as SES and linguistic skills are not considered.

Based on dichotomic comparison, a picture of increased emotional problems, hyperactivity/impulsivity traits and peer relationship difficulties emerged in HB children, compared to the monolingual group. There were no differences in conduct problems and an advantage in prosocial behaviour in HBs, based on parents’ reports. Also, this group received lower scores in the evaluation of emotional and behavioural skills, according to teachers’ reports. Together, in line with previous studies [24, 83, 84], HBs had lower SES and linguistic skills (in the majority language, i.e., Italian), the latter measured either through direct assessment (vocabulary task) or teachers’ evaluations, compared to children from monolingual backgrounds. This first set of results might suggest, in line with previous studies, that HB children might be at higher risk of emotional and behavioural difficulties [41].

However, this representation might oversimplify a complex phenomenon, and the study aimed to delve deeper into this initial picture and add new evidence through an analysis of the differential relationships of language background, together with composite measures of SES and linguistic skills evaluation with emotional and behavioural profile. The five main dimensions of the SDQ questionnaire (parents’ reports) were considered independently, together with teachers’ evaluations of children’s emotional and behavioural skills.

To sum up, moving from dichotomic group analyses to regression models that consider and control the differential role of language background, SES and (Italian) linguistic skills, a more complex pattern of relationships emerged. Linguistic skills were actually found to be the unique concurrent predictor for conduct problems and the dominant predictor for the hyperactivity/impulsivity traits, the problems in peer relationships and the emotional and behavioural skills evaluated by teachers. Also, linguistic skills positively predicted prosocial behaviour. However, linguistic skills were not significantly related to emotional problems as assessed by parents, which had SES as their main predictor instead, followed by language background. There was also a negative significant relationship between lower SES and hyperactivity/impulsivity traits, problems in peer relationships, and prosocial behaviour. Higher SES also predicted better emotional and behavioural skills according to teacher evaluation. Finally, HB background was associated, but not as a primary predictor, with increased emotional problems and peer relationship problems as assessed by parents, and minor emotional and behavioural skills as assessed by teachers. However, it was the main factor to be positively associated with prosocial behaviour.

This picture can be interpreted in light of previous literature, suggesting a main association of L2 linguistic skills with children’s behavioural profiles, with particular emphasis on externalising traits and social relationships [3, 6, 29, 30, 32]. This study was conducted on a typical population, suggesting that the children who have lower L2 linguistic skills might encounter more difficulties in peer relationships and might show increased behavioural problems, possibly due to minor access to verbal communication. SES was found to be primarily associated with emotional problems, in line with previous literature [10, 63–65], suggesting that resources might shape subjective experiences. Differently from what is stated by Peverill et al. [66], findings from the present study seem to suggest a main association with internalising rather than externalising symptoms. Also, SES generally resulted as a non-dominant factor compared to linguistic skills in predicting patterns of socioemotional and behavioural difficulties, suggesting that proximal factors might be prominent over distal factors. This sheds light on the idea that the influence of SES should not be viewed as deterministic or inherent to individuals [16], but rather as a structural variable or a constraint, according to the neuroconstructivist perspective [2], which individuals and society can intervene on. In this regard, previous literature found that the family environment can mediate the influence of SES on children’s early achievements and well-being [18, 85].

Finally, as hypothesised, the strength of the relationship between language background and socioemotional and behavioural problems was less conspicuous, although still present, when L2 linguistic skills and SES were taken into account. As outlined by Araújo Dawson and Williams [36], language status can act as an acculturative stressor throughout the early school experience and can negatively affect well-being. However, an innovative result is increased prosocial behaviours in HB children, whilst lower in high SES children. So, even if HB children might experience a degree of increased difficulty in engaging in peer relationships and might be more at risk for emotional problems, they seem to have active coping strategies that bring them to engage in prosocial behaviours. As previous literature suggests, bilingualism might be associated with both cognitive and everyday life advantages [86, 87].

The study presents some limitations that need to be acknowledged. First of all, results are based on a concurrent design, and this does not allow the definition of causal patterns. As previous evidence suggested [27], bidirectional relationships are also possible, at least in terms of linguistic skills and emotional patterns. It is, instead, more plausible to assume unidirectional patterns of SES and HB to emotional well-being. Secondly, teachers’ evaluation might also be biased by a halo effect that might lead to high correlations between linguistic and behavioural judgments. Also, the teacher’s prejudices against low-SES children could influence their competence assessment. However, correlation analyses highlighted that vocabulary skills were more strongly related to the teachers’ evaluation of linguistic skills than to behavioural items. It has also to be underlined that in the present study we only evaluated linguistic skills in the majority language (Italian), whereas future studies might also include assessment on heritage language. Another point regards the measurement and operationalization of SES. In the present study education and occupation levels from both parents were considered as a composite measure, but other approaches might be adopted [23]. Finally, the present study did not take into account important sources of heterogeneity in the HB sample (e.g., linguistic input and age of exposure to heritage language and L2) [24], and future investigation should also consider positive and negative attitudes toward heritage language in the child and the family [43–47] in order to better investigate under which circumstances HB might be at greater risk of emotional discomfort.

## Conclusions

Despite its limitations, the present study highlights some innovative patterns that might represent new evidence of the differential relationship between SES, linguistic skills and language background on children’s socioemotional and behavioural profiles and might lead to potential implications for research and practice. Linguistic skills (in the majority language, i.e., Italian) and, secondly, SES turned out to be the dominant factors associated with socio-emotional and behavioural well-being. Heritage bilinguals might be at higher risk of emotional discomfort but they also show some advantages in prosocial behaviour, unlike children with higher SES. Finally, the study has been conducted in Italy, where minor evidence has been collected so far on the relationship between the above-cited variables and therefore represents an original contribution to previous literature.

As regards the potential implications, also in line with Singh and Rajendra [69], these results underline the importance of including composite measures of SES in psychological research and suggest that *both* linguistic skills and SES should be included when investigating children’s socioemotional and behavioural well-being, going beyond categorical group comparisons. In terms of practice, it further reinforces the significance of early interventions on linguistic skills in preschool settings. Further, the study highlights the importance of encouraging a multidimensional approach among educators and clinicians when faced with children with emotional and behavioural difficulties, specifically in the case of HB, developing multiple hypotheses on the factors underlying the discomfort and favouring multi-component interventions.

## Data Availability

The data that support the findings of this study are openly available in PsychArchives at https://doi.org/10.23668/psycharchives.14488.
